# Dementia care and the role of guideline adherence in primary care: cross-sectional findings from the DemTab study

**DOI:** 10.1186/s12877-021-02650-8

**Published:** 2021-12-18

**Authors:** Sonia Lech, Julie L. O’Sullivan, Johanna Drewelies, Wolfram Herrmann, Robert P. Spang, Jan-Niklas Voigt-Antons, Johanna Nordheim, Paul Gellert

**Affiliations:** 1 Charité – Universitätsmedizin Berlin, corporate member of Freie Universität Berlin, Humboldt-Universität zu Berlin, and Berlin Institute of Health, Institute for Medical Sociology and Rehabilitation Science, Charitéplatz 1, 10117 Berlin, Germany; 2 Brandenburg Medical School Theodor Fontane, Department of Psychiatry, Psychotherapy and Psychosomatics, Neuruppin, Germany; 3 Department of Psychology, Humboldt Universität zu Berlin, Berlin, Germany; 4 Charité – Universitätsmedizin Berlin, corporate member of Freie Universität Berlin, Humboldt-Universität zu Berlin, and Berlin Institute of Health, Institute of General Practice, Berlin, Germany; 5 Technische Universität Berlin, Quality and Usability Lab, Berlin, Germany; 6 Deutsches Forschungszentrum für Künstliche Intelligenz GmbH (DFKI), Speech and Language Technology, Berlin, Germany

**Keywords:** Dementia, Primary care, Adherence to dementia guideline

## Abstract

**Background:**

General practitioners (GPs) play a key role in the care of people with dementia (PwD). However, the role of the German Dementia Guideline in primary care remains unclear. The main objective of the present study was to examine the role of guideline-based dementia care in general practices.

**Methods:**

A cross-sectional analysis of data obtained from the DemTab study was conducted. Descriptive analyses of sociodemographic and clinical characteristics for GPs (*N* = 28) and PwD (*N* = 91) were conducted. Adherence to the German Dementia Guideline of GPs was measured at the level of PwD. Linear Mixed Models were used to analyze the associations between adherence to the German Dementia Guideline and GP factors at individual (age, years of experience as a GP, frequency of utilization of guideline, perceived usefulness of guideline) and structural (type of practice, total number of patients seen by a participating GP, and total number of PwD seen by a participating GP) levels as well as between adherence to the German Dementia Guideline and PwD’s quality of life.

**Results:**

Self-reported overall adherence of GPs was on average 71% (*SD* = 19.4, range: 25–100). Adherence to specific recommendations varied widely (from 19.2 to 95.3%) and the majority of GPs (79.1%) reported the guideline as only partially or somewhat helpful. Further, we found lower adherence to be significantly associated with higher numbers of patients (γ10 = − 5.58, *CI* = − 10.97, − 0.19, *p* = .04). No association between adherence to the guideline and PwD’s quality of life was found (γ10 = −.86, *CI* = − 4.18, 2.47, *p* = .61).

**Conclusion:**

The present study examined the role of adherence to the German Dementia Guideline recommendations in primary care. Overall, GPs reported high levels of adherence. However, major differences across guideline recommendations were found. Findings highlight the importance of guidelines for the provision of care. Dementia guidelines for GPs need to be better tailored and addressed. Further, structural changes such as more time for PwD may contribute to a sustainable change of dementia care in primary care.

**Trial registration:**

The DemTab trial was prospectively registered with the ISRCTN registry (Trial registration number: ISRCTN15854413). Registered 01 April 2019.

**Supplementary Information:**

The online version contains supplementary material available at 10.1186/s12877-021-02650-8.

## Background

The current and imminent public health impact of dementia is vast. According to the World Alzheimer’s Report published in 2015, 46.8 million people worldwide were estimated to live with dementia. Further, this number is estimated to increase to 74.7 million by 2030 and 131.5 million by 2050 [1, 2]. Currently, about 1.7 million people with dementia (PwD) live in Germany, with a prevalence of 10% among older adults over the age of 65 [3, 4]. Dementia not only affects those living with dementia, but also their families and informal caregivers, the health care system, and society as a whole [5–7]. Consequently, policy makers and researchers are being urged to address dementia as a public health priority. In light of this, the World Health Organization (WHO) has called for national dementia strategies [6]. WHO’s recommendations for national areas of action include, amongst others, the improvement of dementia care delivery. In Germany, general practitioners (GPs) play a pivotal role in the management and delivery of care for PwD [8–13]. For example, almost 99% of PwD living at home consult their GP at least once a year [14]. Despite empirical evidence reporting that GPs acknowledge dementia care as a relevant topic and show positive attitudes towards the care of PwD, GPs find many aspects of dementia care to be challenging [15]. The vast majority of previous research has focused on examining and improving primary care for dementia at an individual level of GPs. For example, research has primarily centered on providing knowledge training and education in diagnostics and dementia management [16–23]. However, structural factors such as time constraints per patient [17, 24], as well as lack of cross-sectional collaboration [25] and lack of social services support [17, 18] were frequently reported to negatively impact primary care delivery for dementia. It remains unclear which GP related factors impact dementia care delivery most.

Overall, evidence-based guidelines represent one public health tool that fosters optimal care delivery [26]. Following recommendations of evidence-based guidelines may contribute to an improvement of dementia care [27, 28] and patient health-related quality of life [29]. In Germany, the German Dementia Guideline (GDG) [28] provides evidence-based recommendations for treatment, care, and support of dementia. The GDG is an interdisciplinary guideline which is jointly issued by the German medical society for neurology, and the German medical society for psychiatry, psychotherapy, and psychosomatics. This comprehensive guideline comprises information on state-of-the-art diagnosis of Alzheimer’s disease and other dementias as well as evidence-based recommendations for pharmacological and psychosocial treatment of dementia. Depending on dementia severity, recommendations are given for treatment of the core symptoms of dementia, including cognitive, functional, and behavioral symptoms. For example, the GDG recommends an intake of anti-dementia drugs dependent on type of dementia and severity of cognitive impairment. For individuals diagnosed with Alzheimer’s Disease and a mild to moderate cognitive impairment, the intake of Acetylcholinesterase inhibitors is recommended [28]. The guideline also includes information on caregiver burden and specific health risks for informal caregivers and provides recommendations on interventions for reducing their psychological burden. Regarding dementia treatment and care in the primary care setting, the guideline contains a specific chapter with information on the unique role of GPs which was added by the German College of General Practitioners and Family Physicians (DEGAM). The chapter outlines the importance of a holistic view and, in the best sense of participatory decision-making, recommends to prioritize the individual health status and health problems of patients.

While the implementation of and adherence to dementia guideline recommendations may improve dementia care, little is known about the knowledge and utilization of the GDG among GPs in Germany. However, the GDG was not specifically developed for general practice. In addition, the associations between adherence to dementia guidelines (AGDG) and GP and PwD related factors remain unclear. The present study aims to explore the role of using the GDG in recommendations in primary care. First, we aim at describing a newly developed checklist assessing adherence to the GDG. Second, we aim to examine the association between AGDG and GP factors at *individual levels* (age, years of experience as a GP, frequency of utilization of the GDG, and perceived usefulness of the GDG) as well as at *structural levels* (type of practice, total number of patients seen by a participating GP during last 3 months, and total number of PwD seen by a participating GP during last 3 months). Based on previous literature [29], the following hypothesis are proposed:

Hypothesis 1: Structural factors (type of practice, total number of patients seen by a participating GP during last 3 months, and total number of PwD seen by a participating GP during last 3 months) will have a greater impact on AGDG than individual factors (age, years of experience as a GP, frequency of utilization of the GDG, and perceived usefulness of the GDG).

Hypothesis 2: The AGDG score will be positively associated with PwD’s self-reported quality of life.

## Methods

### Participants and recruitment

This paper uses baseline data obtained from the DemTab study, a cluster randomized controlled trial (cRCT) that investigated the effects of a tablet-based intervention on guideline adherence (primary outcome) and health related PwD and informal caregiver outcomes (secondary outcomes) in the primary care setting. The study design and methods for the DemTab study have been published in detail elsewhere [30]. In summary, the target population of the DemTab study were GPs, PwD, and their informal caregivers from Berlin and the surrounding area and data was obtained from GPs, PwD and their informal caregivers. For the purpose of the present study, only baseline data from GPs and PwD were included. Eligible GPs were currently operating GPs who provided informed consent to participate in the study. Eligible PwD were community living patients with a dementia diagnosis (ICD-10 F00-F03, G30, G31.0 and G31.82), who were treated in outpatient care and provided a signed informed consent to participate in the study (if he/she is still authorized to sign) or otherwise through a person holding the power of attorney.

### Study sample

**Fig. 1:**
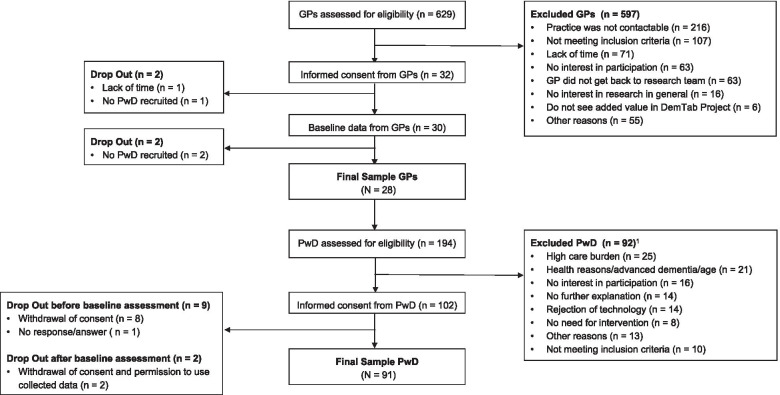
Flow chart of recruitment. Note: ^1^ A total of *N* = 92 PwD were not included in the study. The number provided to describe reasons for non-participation (*n* = 111) are not equal with the sum of excluded PwD, as *n* = 20 reported multiple reasons for non-participation

Due to the cluster-randomized design, the study sample was determined in two steps. In the first step, GPs were recruited through a variety of sampling methods: 1) advertisements in general practice related publications and newsletters through different networks in and around Berlin; 2) via phone recruitment of GPs randomly drawn from a database of the Statutory Health Insurance Physicians in Berlin, and 3) face-to-face recruitment of GPs in their general practices in Berlin. In a second step, successfully recruited GPs recruited potentially eligible PwD from their practice. Overall, 629 GPs and 194 PwD were contacted for recruitment, of which 32 GPs and 102 PwD agreed to participate and signed an informed consent. On average, each GP referred about 7 PwD (range: 1–17; mdn = 6; IQR = 3.5) and successfully recruited about 4 PwD (range: 1–11; mdn = 3; IQR = 3.5). The final sample consisted of *N* = 28 GPs and *N* = 91 PwD. A flowchart is presented in Fig. 1. A thorough description of the recruitment process and responses rates can be found in Lech et al. [31].

### Data collection

Baseline data were collected from July 2019 to July 2020. Data from GPs were obtained through a questionnaire sent via mail. Data collection from PwD was originally planned and in most cases obtained by trained study nurses in the patient’s home. However, due to the coronavirus disease 2019 (COVID-19), data collection was aligned with new regulations and changed from face-to-face assessment to phone interviews (*n* = 12 PwD). The first assessment via phone was conducted on 30th March 2020. With the exception of the Mini Mental State Examination (MMSE), data collection via phone was uncomplicated and feasible. A follow up analysis revealed no differences in variables of PwD between data collected via face to face and data collected via phone interviews. However, due to the adjusted baseline data collection, it was not feasible to obtain data on the MMSE in a total of 11% of PwD (*n =* 11). Additional information on the health and care situation of each PwD was obtained from GPs via another questionnaire.

### Measures

At baseline, variables of interest were collected using a self-report questionnaire. A detailed description of all variables and measures can be found elsewhere [30]. The DemTab study, with respect to autonomy and self-determination, aimed at involving and letting PwD speak for themselves as much as possible during data collection. Therefore, we mainly selected self-reported standardized measurements suitable for PwD. All further information (mainly sociodemographic information) was intended to be obtained from PwD directly. However, if the PwD was no longer able to provide answers or the validity of answers was questionable, a trained study nurse verified or obtained this information from the informal caregiver. For example, if a PwD was unable to provide information on their age or seemed unsure, the study nurse noted this during data collection and afterwards tried to verify the missing information with the caregiver. Study nurses always documented whether sociodemographic information was collected only from the PwD or also from the caregiver. In a total of 61.5% additional data on PwD was obtained from informal caregivers.

#### Measures of adherence to German dementia guideline

Adherence to the German Dementia Guideline (AGDG) was primarily assessed with a 23-item checklist. The checklist was developed based on the German Dementia Guideline [28] and other empirical work focusing on the role of guideline-based primary care [29, 32, 33]. AGDG was self-reported by each GP on patient’s level (for each participating PwD). The checklist can be found in German (original) and English (simple translation) in Appendix 1. The original checklist was composed in a dichotomous format with “yes” and “no” as options, but also included the category “not applicable”. However, when analyzing the data, it became evident that the category “not applicable” was selected inconsistently. Specifically, because we failed to define “not applicable” a priori, it was unclear how this category was used. Consequently, there were known inconsistencies. In order to analyze the impact of the category “not applicable” and reduce possible bias in the calculation of the final AGDG score, we conducted a set of analyses to compare different scorings (see Appendix 2; Table A1). *Scoring method 1:* “not applicable” was recoded into missing data. *Scoring method 2:* “not applicable” was recoded into “not guideline adherent” (= 0), *Scoring method 3:* items, where “not applicable” was plausible were recoded into “not guideline adherent” (= 0), all other “not applicable” were recoded into missing data. *Scoring method 4:* items, where “not applicable” was plausible were recorded into “guideline adherent” (= 1), all other “not applicable” were recoded into missing data. For each scoring method, means and final scores were calculated (see Appendix 2; Table A1). Comparisons of means and correlations across scoring methods did not reveal any significant differences (see Appendix 2; Table A2 and Table A3). Due to conceptual assumptions, *scoring method 1* was chosen for the calculation of the final score and “not applicable” was recoded as missing data. It is recommended for future research, when applying the present or any checklist for the assessment of guideline adherence, to define and include the category “not applicable” when appropriate, as this category may represent a valuable contribution. The final AGDG score for each PwD was calculated as the proportion of guideline adherence and all items answered ([sum of items answered as guideline adherent/sum of all answered items] × 100). The overall AGDG score was calculated as the mean percentage of per-patient guideline adherence across all GPs. The internal consistency of our scale for this data was Cronbachs‘s α = .876.

In addition, we assessed other indicators measuring adherence to the GDG in primary care. First, *knowledge of the guideline* (“*Are you familiar with the dementia guideline?*”; yes/no), *utilization of the guideline* (“*Do you use the dementia guideline?*”, yes/no), *frequency of utilization* (“*How often do you use the guideline*?”; always/often/sometimes/seldom/never) and *perceived usefulness of the guideline* (“*How useful do you find the guideline?”;* very/partially/somewhat/not helpful at all) were assessed from GPs. Further, *prescribed anti-dementia drug* (drug name), *type of dementia* (ICD-10 code) and *cognitive status* (MMSE) were compared based on guideline recommendations and a variable was computed (0 = not guideline adherent, 1 = guideline adherent, 2 = off-label use) to assess guideline adherence with regard to drug prescriptions.

#### Measures of GPs and PwD

Next, demographic and practice information was also collected for GPs. This information included *age* (years), *gender* (female/male/other), *years of experience* as a GP (years), *type of practice* (single/shared), *total number of patients seen by a participating GP during last 3 months* (NPAT) and *total number of PwD seen by a participating GP during last 3 months* (NPWD).

Finally, sociodemographic information of PwD were collected, including *age* (years), *gender* (female/male/other), *education* (years of education) and *living situation* (alone/with partner/with caregiver/in outpatient facility). Further, *level of care was measured according to the compulsory long-term care insurance in Germany* (ranging from 1 = low level of care to 5 = high level of care) [34]. Information on *diagnostic procedure* (“*Who diagnosed the patient?*”; current GP/other GP/specialist/other facility), *type of dementia* (ICD-10 code) and *prescribed medications* were obtained via GPs. Dementia related assessments included the *Mini-Mental State Examination* (total score ranges from 0 to 30, higher scores indicating higher cognitive status) [35]. Quality of Life was assessed using the *Quality of Life in Alzheimer’s Disease questionnaire (QOL-AD,* total score ranges from 13 to 52, higher scores indicating better quality of life) [36].

### Statistical analysis

First, descriptive analyses (means, standard deviations and ranges for continuous variables, frequencies for nominal and ordinal variables) of sociodemographic and clinical characteristics for GPs and PwD as well as for AGDG were calculated. Second, to address Hypothesis 1 and Hypothesis 2, Linear Mixed Models (LMM) for continuous outcomes (covariance type = variance components, estimation = Maximum Likelihood) were applied to analyze the predictive values of independent variables (level 1) accounting for the nested structure (GPs, level 2). The ID of GPs was used as a clustering variable. In step 1, an intercept-only model (no level-one or level-two predictor was included in the model) was estimated to examine the variance associated between GP units and AGDG (base model). In order to describe dependencies due to the cluster structure of the data, an intraclass correlation coefficient (ICC) representing the ratio of the between-GP variance to the total variance was calculated. In step 2, a two-level random-intercept model, which allows for variation in intercepts across GPs was estimated, in order to account for the clustered structure of the data. In order to explore the association between individual and structural factors and AGDG (Hypothesis 1), the following predictors were included in this model: 1) individual factors*: age, years of experience as a GP*, *frequency of utilization of guideline* and *perceived usefulness of guideline,* and *2)* structural factors: *type of practice*, *NPAT* and *NPWD.* In order to examine the association between PwD’s quality of life and AGDG (Hypothesis 2), quality of life was included as a predictor variable in another model. All predictors were standardized. The likelihood ratio (LR) test was used to compare the difference between the two nested models. All statistical analyses were performed using IBM SPSS Statistics for Windows V.27.0 and RStudio (Version 1.4.1106). All tests of significance were based on a *p* < .05 level and confidence interval of 95%.

## Results

### Characteristics of GPs

Characteristics of participating GPs can be found in Table 1. Overall, 61.0% of participating GPs were female and on average 50 years old (*SD* = 7.99, range: 38–67), with a mean of about 12 years of experience as a GP (*SD* = 9.11, range: 1–29). Less than half of GPs (*n* = 12, 42.9%) were working in a single-handed practice. On average, GPs treated *N* = 1489 patients (*SD* = 656.03, range: 700–2990) and *N* = 61 PwD (*SD* = 52.80, range: 9–200) during the last 3 months.

**Table 1:** Main characteristics of GPs

*Sociodemographic characteristics*	*N*	%	*M*	*SD*	range
Age	28		49.9	8.0	38–67
Gender (female)	17	60.7			
Years of experience as a GP	28		11.8	9.1	1–29
Single-handed practice (yes)	12	42.9			
NPAT	28		1488.9	656.0	700–2990
NPWD	28		60.9	52.8	9–200

*N* = 28 GPs, *M* Mean, *SD* Standard Deviation, *NPAT* total number of patients seen by a participating GP during last 3 months, *NPWD* total number of PwD seen by a participating GP during last 3 months

### Characteristics of PwD

Table 2 presents an overview of PwD’s main characteristics. Overall, almost 60% (*n* = 54) of PwD were female, were on average 80 years old (*SD* = 6.3, range: 63–94), and reported an average of 12.6 years of education (*SD* = 3.3, range: 8–17). More than half of PwD lived together with their spouse or partner (*n* = 53; 58.2%). About 51% of PwD were in need of substantial care (care level 3 or higher). About half of PwD obtained their dementia diagnosis from a specialist (55.2%) and about a third (33.3%) from a GP. PwD visited their GP on average 2.8 times in the last 3 months (*SD* = 1.9, range: 0–11). The mean MMSE score was 18.9 (*SD* = 7.8, range: 0–30) and the majority of PwD (*n* = 38; 51.4%) were mildly cognitive impaired. More than one third of PwD (36.7%) reported the intake of an anti-dementia drug. The mean QOL-AD score was 34.1 (*SD* = 5.8, range: 18–48).

**Table 2:** Main characteristics of PwD

*Sociodemographic characteristics*	*n*	%	*M*	*SD*	range
Age	91		80.5	6.3	63–94
Gender (female)	54	59.3			
Years of education	85		12.7	2.8	8–17
Living situation					
Alone	17	18.7			
With spouse/partner	53	58.2			
With another informal caregiver	6	6.6			
In outpatient facility	15	16.5			
Care level (yes)	71	64.6			
Care level 1	5	5.5			
Care level 2	20	22.0			
Care level 3	29	31.9			
Care level 4 or 5	17	18.7			
*Dementia related assessments*					
Diagnostic procedure					
Current GP	16	18.4			
Other GP	13	14.9			
Ambulatory specialist (psychiatrist, neurologist)	48	55.2			
Other facility	10	11.4			
Type of dementia diagnosis					
Alzheimer’s Disease	34	37.4			
Unspecified dementia	32	35.2			
Vascular dementia	17	18.7			
Other type of dementia diagnosis	7	7.7			
MMSE score	74		18.9	7.8	0–30
Severity of cognitive impairment					
Mild	38	51.4			
Moderate	27	36.5			
Severe	9	12.2			
Intake of anti-dementia drugs (yes)	33	36.7			
QOL-AD	91		34.1	5.8	18–48

*N* = 91, *M* Mean, *SD* Standard Deviation, *MMSE* Mini Mental State Examination, *QOL-AD* Quality of Life in Alzheimer’s Disease questionnaire

### The role of the GDG in primary care

The overall mean AGDG score was 71.02 (*SD* = 19.4, range: 25–100). Table 3 shows frequencies for each recommendation of the GDG across all GPs and PwD.

**Table 3:** Guideline adherence over all PwD on item level

Items of the checklist	*n*^*a*^	Yes^b^ (%)
Was a basic geriatric assessment conducted?	88	93.2
Which of the following diagnostic examinations were conducted?		
Physical examination and psychopathological/psychiatric evaluation	89	94.3
Laboratory tests	88	94.3
Differential diagnostics	84	76.2
Cognitive and neuropsychological tests	84	78.6
Recent medical history	83	74.7
CT/MRI scans	81	72.8
Did the GP administer a cognitive screening test?	85	54.1
Were further physical impairments/medical conditions assessed?	90	87.8
Were further mental health impairments/psychiatric conditions assessed?	89	74.2
Did the PwD/family caregiver receive advice concerning psychological and behavioral symptoms of dementia?	82	91.5
Was the entire current medication assessed and discussed?	85	95.3
Were pharmacological treatment options for dementia discussed with the PwD/family caregiver?	81	65.4
Were non-pharmacological interventions for dementia discussed with the PwD/family caregiver?	84	72.6
Were non-pharmacological interventions recommended or prescribed?	84	54.8
Is the PwD currently being treated by a dementia specialist?	84	61.9
Were further care services for PwD discussed with the PwD/family caregiver?	84	64.3
Was the PwD/family caregiver informed about local support services for PwD?	75	54.7
Was a care plan developed with the PwD/family caregiver?	82	54.9
Were daily activities and how to maintain them discussed with the PwD/family caregiver?	82	79.3
Were self-help measures discussed with the PwD/family caregiver?	84	69.0
Were newly emerging risks assessed and discussed?	80	62.5
Were driving skills or lack thereof discussed with the PwD/family caregiver?	59	49.2
Was the PwD/family caregiver approached about an application for a care level from the German nursing care insurance?	79	79.7
Was the PwD/family caregiver made aware of their rights and the availability of local advocacy services?	81	67.9
Was palliative care discussed?	73	19.2
Was the caregiver stress level discussed in detail with the family caregiver?	81	74.1
Was the family caregiver informed about support offers for family caregivers?	79	87.1

*N* = 28 GP, Adherence to German Dementia Guideline was self-reported by each GP on patient’s level (for each participating PwD), n^a^ = total number of PwD for whom adherence to the specific recommendation (item) was rated by the treating GP, Yes^b^ = percentage of PwD for whom treating GPs reported following a specific recommendation (=being adherent to guideline recommendation)

The great majority of GPs reported following the guideline recommendations with regard to assessing a patient’s entire current medication plan (95.3%), physical and psychopathological evaluations (94.3%), laboratory tests as part of the diagnostics procedure (94.3%), conducting a basic geriatric assessment (93.2%), and assessing psychological and behavioral symptoms of dementia (91.5%). Recommendations on discussing palliative care (19.2%) or the current driving situation (49.2%), as well as obtaining CT/MRI scans as part of the diagnostic procedure (54.1%), providing of information about local support services (54.8%) and prescribing non-pharmacological interventions (54.8%) were less frequently followed. Further, the great majority (*n* = 20; 71.4%) of GPs reported to be familiar with the GDG, but only 19.2% (*n* = 5) reported using it often. Further, 20.8% (*n* = 5) reported the GDG as very helpful and 45.8% (*n* = 11) found it to be partially helpful. Almost one third (*n* = 8; 28.6%) reported the length of the GDG as a reason for not using the guideline. See Table 4 for a complete breakdown of attitudes toward the GDG. With regard to anti-dementia drug prescription, 10.3% of PwD were prescribed a drug that was not in line with guideline recommendations, and 44.8% were prescribed a drug that was considered as off-label use by the GDG.

**Table 4:** GPs attitudes toward the German Dementia Guideline

*Attitudes*	*n*	%
Knowledge about guideline (yes)	20	71.4
Utilization of guideline (yes)	17	60.7
Frequency of utilization	26	
Often	5	19.2
Sometimes	10	35.7
Seldom	7	26.9
Never	4	15.4
Helpfulness of guideline	24	
Very	5	20.8
Partially	11	45.8
Somewhat	8	33.3
Not helpful at all	0	0
Reasons for non-utilization of guideline	*14*	
Length	8	28.6
Lack of relevance	4	14.3
Lack of knowledge	2	7.1

*N* = 28 GPs

### Association between AGDG and factors on GP and PwD level

Results of the intercept-only model (base model) indicated there was statistically significant variation in the intercepts (ICC = .536), accounting for approximately 54% of the variance in AGDG and indicating a substantial clustering of observations within level 2 units. With regard to Hypothesis 1, the regression coefficient for NPAT showed a negative and significant predictive relationship between NPAT and AGDG (γ10 = − 5.58, *CI* = − 10.97, − 0.19, *p* = .04), indicating an association between higher number of patients and lower AGDG scores. *Age* (γ10 = − 7.39, *CI* = − 19.81, 5.03, *p* = .23), *Years of experience as a GP* (γ10 = 7.92, *CI* = − 5.03, 20.86, *p* = .22), *frequency of utilization of GDG* (γ10 = − 2.06, *CI* = − 12.05, 7.93, *p* = .68) and *perceived usefulness of GDG* (γ10 = 2.78, *CI* = − 5.71, 11.29, *p* = .51) as well as *type of practice* (γ10 = − 2.54, *CI* = − 7.73, 2.65, *p* = .33) and *NPWD* (γ10 = − 3.26, *CI* = − 9.06, 2.53, *p* = .26) were not significant in predicting AGDG. Further, results of the likelihood ratio test showed a significant increase of the fit by adding level 1 predictors (*χ*^*2*^ = 155.6, *df* = 7, *p* < .001). With regard to Hypothesis 2, the regression coefficient for QOL-AD shows no significant association between QOL-AD and AGDG score (γ10 = −.86, *CI* = − 4.18, 2.47, *p* = .61).

## Discussion

The main objective of the present study was to examine the role of the German Dementia Guideline in primary care. The main objective of the present study was to examine the role of the German Dementia Guideline in primary care. Previous research has already acknowledged the central role of GPs in diagnostics, treatment and care of dementia. Generally, results of the present study underline the key role of GPs in dementia care. For example, in the present study, more than one third of PwD received their dementia diagnosis from a GP. This finding is in line with recent empirical data from Germany [37]. Further, findings of the present study indicate overall high levels of AGDG, although large differences can be observed across recommendations. With regard to Hypothesis 1, the total number of patients seen by a participating GP during the last 3 months was significantly and negatively associated with AGDG. With regard to Hypothesis 2, quality of life was not significantly associated with AGDG.

### Adherence to the German dementia guideline in primary care

For the purpose of this study, a checklist was developed to examine the role of adherence to the German Dementia Guideline. This checklist facilitates the assessment of AGDG for research (calculation of AGDG score) and may assist GPs in daily practice with treatment and care of dementia. With regard to the AGDG score, present findings indicate a relatively high overall guideline adherence among participating GPs. In contrast to our study, a study examining the effect of a disease management intervention on guideline adherence reported a much lower overall mean score of guideline adherence [29]. However, Vickrey et al. (2006) obtained information on guideline adherence by medical record review as well as by caregiver survey. The present study measured AGDG primarily with a self-report checklist. While the overall AGDG was relatively high, variability between recommendations were found. With regard to palliative care, past research has frequently acknowledged, that due to the progressive nature of dementia, advance care planning and palliative care is important, and GPs play a key role in the in-time planning [38–40]. In order to ensure and respect preferences and wishes of PwD, it is recommended to ascertain their views in an early stage of the disease, before ability to consider the future is limited [41]. As the majority of community dwelling PwD receive regular care from their GPs and GPs often have a longstanding relationship with their PwD, GPs are particularly suited to address palliative care [42]. In order to improve advanced and palliative care planning, we recommend that dementia guidelines should include guidance and recommendations on that matter. With regard to the present result on fitness to driving, the GDG specifically provides a section on dementia and driving and outlines the importance of evaluating current driving skills with the progression of dementia [28]. Previous research has acknowledged, that GPs play a key role in the assessment of fitness to drive in dementia, a topic of uncertainty and conflict for GPs [43]. A recent study found that GPs discussed fitness to drive with only 32.1% of potentially driving elderly patients [44]. Previous studies indicated that fitness to drive is severely impaired in moderate and severe dementia [45]. In sum, there is an urgent need to develop and provide training and guidance on performance of driving assessments for GPs so that they are able to perform such assessments with PwD [46].

With regard to the AGDG score, it is important to discuss the interpretation of the score. The aim of the present checklist was to examine and measure adherence to the GDG recommendations among GPs. Previous empirical work has acknowledged, that evidence-based guidelines may contribute to an improvement of care provision [26–28]. Building on this, a checklist was developed based on the recommendations of the current GDG. However, adherence to the GDG does not necessarily indicate best quality of care provided for individuals. As stated in the GDG recommendations of the DEGAM, a holistic view on PwD as well as provision of individualized medicine based on the current (health) needs of individuals is of great importance. With regard to the necessity of individualized treatment options especially for patients with multimorbidity, lower adherence to the recommendations still may propose better care provision for a given individual. However, awareness of evidence-based guidelines, knowledge about specific guideline recommendations and provision of care based on shared decision-making represent basic requirements for individualized care. We believe that the proposed checklist may serve as an overview of the most important aspects of dementia care with the aim to facilitate knowledge transfer, to support GPs in their decision-making and care provision and to allow GPs to assess and evaluate their adherence to specific guideline recommendations. Therefore, the checklist can be of great value, especially for practitioners. However, the present checklist does not take into account the provision of individualized care for PwD in primary care nor represent the quality of care provided by GPs. Especially in primary care, where GPs have many years of knowledge about their patients and their individual environments, (health) needs and preferences, deviations from specific guideline recommendations must be recognized in order to facilitate the provision of individualized treatment and optimal care. In addition to the AGDG score, we have analyzed data on anti-dementia drug intake with regard to guideline adherence. The GDG recommends intake of anti-dementia drugs dependent on type of dementia and severity of cognitive impairment. For example, for individuals diagnosed with Alzheimer’s Disease and a mild to moderate cognitive impairment, the intake of Acetylcholinesterase inhibitors is recommended [28]. In the present sample, about 37% of PwD reported the intake of an anti-dementia drug, a finding in line with previous studies [47, 48]. For example, a study on medical treatment of PwD in Germany reported 25% of ambulatory PwD receiving an anti-dementia drug, and found that this number varied depending on whether PwD were seen by a GP and specialist or solely a GP (48% versus 24.5%, respectively) [49]. Past research has consistently reported a positive association between involvement of a GP/specialist and anti-dementia drug prescription [48, 50]. In the present study, about 10% of PwD reported the intake of an anti-dementia drug which was not in line with GDG recommendations, and almost half (44.5%) reported an intake of off-label drugs. With regard to medication, based on present findings, the prescription of anti-dementia drugs requires improvement. An anti-dementia drug treatment should be always based on individual assessments of risks and benefits [28, 47]. Key dementia care providers, especially GPs, should have knowledge on the latest guideline recommendations regarding anti-dementia drugs and their risks and benefits. Collaborative care models may improve anti-dementia drug prescriptions in ambulatory care for PwD. Our data shows that the majority of PwD (62%) saw a specialist in addition to their GP. Collaborations between GPs and specialists (e.g., psychiatrist or neurologist) can improve the implementation of guideline recommendations with regard to anti-dementia drugs [47, 51–54].

Finally, nearly one third of GPs who participated in this study reported length of the GDG as a reason for non-utilization of the guideline and another third of GPs reported the guideline as only somewhat helpful. Given these findings, research should reconsider the current format of the GDG for GPs. More compiled and practical guidelines are needed. Further, it is of great importance to include perspectives and recommendations from general practice in the guideline development. Although the GDG acknowledged the important role of GPs in the care of PwD [28], the German College of General Practitioners and Family Physicians was hardly involved in the development of the guideline. The validity of the current GDG expired in February 2021. Thus, a new guideline is currently being developed. We highly recommend including the perspectives and experiences of GPs in the development and implementation of the new GDG.

### Associations between AGDG and variables on GP and PwD level

It was of great interest to examine the associations between AGDG and factors on GPs and PwD level. With regard to individual and structural factors of GPs and AGDG, results of multilevel analyses revealed that only the total number of patients seen by a participating GP during the last 3 months were negatively associated with AGDG. This finding is partially in line with previous empirical work. While it is widely believed that more time per patient improves patient’s health and quality of care [55–57], a systematic review of clinical trials found insufficient empirical evidence that patients benefit from longer consultations [58]. However, with regard to dementia, past research has recommended more time in primary care for PwD [12]. In Germany, a recent study found an average consultation length of 7.6 min [59]. It is reasonable to believe that GPs with larger patient loads have less time to spend with each patient, consequently resulting in less time to focus on and follow guideline recommendations. However, the observed effect should be interpreted with caution. The present study has no data on the frequency of visits for each patient nor the total number of hours GPs actually spend with their patients. Future research is needed in order to gain a better understanding of the role of a GPs patient load, time spent with each patient, and time spent on patient care, and its impact on guideline adherence.

With regard to the association between AGDG and PwD’s quality of life, no significant association was found. This finding is not consistent with previous research that examined the effects of a dementia guideline-based disease management program in a cRCT and found significant improvements in health-related quality of life in PwDs [29, 60]. Future research is required in order to gain a better understanding of the role of guideline-based dementia care in primary practices [61]. The present paper is based on baseline data collected within a cRCT that aims to evaluate the effect of a technology-based intervention on AGDG in primary care. We are currently conducting follow-up assessments with GPs and PwD and will be able to conduct a more in-depth examination of the association between AGDG and GP and PwD in the near future.

### Limitations

This is the first study in Germany assessing adherence to the German Dementia Guideline in primary care with a checklist developed based on the GDG recommendations. However, there is a number of limitations that must be outlined. First, the present sample is drawn from a cRCT examining a tablet-based intervention for GPs, PwD, and their informal caregivers. The DemTab study is based on a convenience sample. Hence, in the present study GPs, PwD and their caregivers self-selected themselves into the DemTab study. The so-called self-selection may propose a higher risk of biased data. Participants’ decision to participate may be correlated with traits that affect the study [62]. For example, the high guideline adherence found in our study may be because participating GPs were particularly engaged and interested in the study’s topic. Further, as PwD agreed to use a technological device as part of the intervention, it may be that participating PwD were of greater health compared to a general sample of PwD. The self-selection bias is a known problem in research [63]. For example, Keiding & Louis (2016) argue that self-selection directly affects the validity of cross-sectional analyses and longitudinal trends [64]. This limitation must be taken into account when interpreting results. Based on the recruitment strategies of the present study, which were conducted in line with data protection laws, self-selection of PwD was hard to prevent, as contact information of patients was only forwarded by GPs once PwD agreed to it. However, the potential influences of self-selection for study participation were mitigated by strict inclusion and exclusion criteria. Only a subset of participants who wanted to participate in the study was selected for participation. Second, regarding the assessment of guideline adherence, the post hoc recoding of the category “not applicable” as missing data must be addressed. Even if comparisons of means and correlations across scoring methods did not reveal any significant differences, data labeled as “not applicable” can provide further insight into responses and, therefore, may represent a valuable contribution in the assessment and the interpretation of AGDG. Adherence to a specific guideline recommendation may not be applicable for some individuals due to patient related (health) reasons (for example risk of side effects with regard to polypharmacy), GP related reasons (for example expert knowledge and personal appraisal) or structural reasons (for example lack of access to specific health services). Hence, there may be various reasons why a specific guideline recommendation is not applicable for a given individual. The current AGDG score does not account for that. This limitation must be considered when interpreting the AGDG score. Future research, when using the present or any checklist to assess guideline adherence, should define and include this category in order to improve the validity and explanatory power of the score. Third, with regard to the checklist, guideline adherence in the present study was measured with a self-report checklist. A potential rater bias (systematic introduction of variance by GPs) has to be kept in mind when interpreting the scores of guideline adherence in the present study [65, 66]. Future research should assess guideline adherence in a more objective manner. Last, with regard to Hypothesis 1 and Hypothesis 2, due to the cross-sectional design we cannot draw causal relationships. Future studies should examine longitudinal data in order to examine multidirectional associations between guideline adherence and GP and PwD related variables.

## Conclusion

The present study examined the role of adherence to the German Dementia Guideline provided by GPs in Germany. Overall adherence to the guideline was relatively high. However, major differences between recommendations were observed. In order to ensure and improve primary care of PwD, dementia guidelines for GPs need to be better tailored and addressed. Further, collaborative care between GPs and specialists as well as structural changes, such as GPs spending more patient time with PwD, may contribute to a sustainable change of dementia care in primary care.

## Supplementary Information

**Additional file 1.** Appendix 1.

**Additional file 2.** Appendix 2.

## Data Availability

Data is stored in a non-publicly available repository. Data are however available from the corresponding author on request.

## Abbreviations

AGDG: Adherence to dementia guideline
cRCT: Cluster randomized trial
GDG: German Dementia Guideline
GP: General practitioner
ICC: Interclass correlation coefficient
Mdn: Median
MMSE: Mini Mental State Examination
NPAT: Total number of patients seen by a participating GP during last 3 months
NPWD: Total number of PwD seen by a participating GP during last 3 months
PwD: People with dementia
SD: Standard deviation
UK: United Kingdom
QOL-AD: Quality of Life in Alzheimer’s Disease questionnaire
