# Supplementary material for: Dementia care and the role of guideline adherence in primary care: cross-sectional findings from the DemTab study
Source: BMC Geriatr. 2021 Dec 18;21:717. doi: 10.1186/s12877-021-02650-8 (PMC8683809; doi:10.1186/s12877-021-02650-8)
Supplement: Supplementary file 1 — Additional file 1. Appendix 1. [file 12877_2021_2650_MOESM1_ESM.pdf]

Appendix 1

Checklist for GPs in German (Original)

Frage	ja	nein	nicht zutreffend
Wurde ein geriatrisches Basisassessment durchgeführt?			
Welche der folgenden Untersuchungen wurden im Rahmen der Diagnostik durchgeführt:<br><br>Körperliche und psychopathologische Untersuchung<br>Labor-Diagnostik<br>Differenzialdiagnostik<br>Schweregradabschätzung/Kognitive Testung<br>Verlaufsuntersuchung<br>CCT/fMRT			
Wurde ein kognitives Screening in der Hausarztpraxis durchgeführt?			
Wurden weitere körperliche Beeinträchtigungen/Erkrankungen erfasst?			
Wurden weitere psychische Beeinträchtigungen/Erkrankungen erfasst?			
Wurden mit dem Patienten/Angehörigen psychische und Verhaltenssymptome der Demenz ausführlich besprochen?			
Wurde die gesamte aktuelle Medikamenteneinnahme erhoben und besprochen?			
Wurde eine medikamentöse Therapie der Demenz mit dem Patienten/Angehörigen besprochen?			
Wurden nicht-medikamentöse Therapieangebote der Demenz mit dem Patienten/Angehörigen besprochen?			
Wurden dem Patienten nichtmedikamentöse Therapien empfohlen oder verordnet?<br>Wenn ja, welche________________________________			
Befindet sich der Patient aufgrund der Demenz in fachärztlicher Behandlung?			
Wurden mit dem Patienten/Angehörigen weitere Versorgungsangebote besprochen?			
Wurde der Patient/Angehöriger über niedrigschwellige Angebote informiert?			
Wurde ein Behandlungsplan zusammen mit dem Patienten/Angehörigen erstellt?			
Wurden mit dem Patienten/Angehörigen Alltagsgestaltung und -kompetenzen besprochen?			
Wurden mit dem Patienten/Angehörigen eigentherapeutische Maßnahmen besprochen?			
Wurden neu aufgetretene Risiken besprochen? (z.B. Selbst-, Fremdgefährdung)			
Wurde mit dem Patienten/Angehörigen die Fahrtauglichkeit besprochen?			
Wurde der Patient/Angehörige auf die Beantragung eines Pflegegrades angesprochen?			
Wurde der Patient/Angehörige auf rechtliche Vorsorgemaßnahmen angesprochen?			
Wurde über Palliativversorgung gesprochen?			
Wurde die Belastungssituation des Angehörigen ausführlich besprochen?			
Wurde der Angehörige über Entlastungs- und Beratungsangebote informiert?			

Checklist for GPs in English (simple translation)

Question	yes	no	not applicable
Was a basic geriatric assessment (geriatrisches Basisassessment) conducted?			
Which of the following examinations were conducted during the diagnostic process?			
Physical examination and psychopathological/psychiatric evaluation			
Laboratory tests			
Differential diagnostics			
Cognitive and neuropsychological tests			
Recent medical history			
CT/MRI scans			
Did the GP administer a cognitive screening test?			
Were further physical impairments/medical conditions assessed?			
Were further mental health impairments/psychiatric conditions assessed?			
Did the patient/family caregiver receive advice concerning psychological and behavioral symptoms of dementia?			
Was the entire current medication assessed and discussed?			
Were pharmacological treatment options for dementia discussed with the patient/family caregiver?			
Were non-pharmacological interventions for dementia discussed with the patient/family caregiver?			
Were non-pharmacological interventions for dementia recommended or prescribed?<br>If yes, which intervention_________________________________			
Is the patient currently being treated by a dementia specialist (i.e., neurologist, geriatric psychiatrist)?			
Were further care services for people with dementia discussed with the patient/family caregiver?			
Was the patient/family caregiver informed about local support services for people with dementia?			
Was a care plan developed with the patient/family caregiver?			
Were daily activities and how to maintain them discussed with the patient/family caregiver?			
Were self-help measures discussed with the patient/family caregiver?			
Were newly emerging risks assessed and discussed? (i.e., self-harm or harming others)			
Were driving skills or lack thereof discussed with the patient/family caregiver?			
Was the patient/family caregiver approached about an application for a care level from the German nursing care insurance (Pflegegrad)?			
Was the patient/family caregiver made aware of their rights and the availability of local advocacy services?			
Was palliative care discussed?			
Was the caregiver stress level discussed in detail with the family caregiver?			
Was the family caregiver informed about available resources and support offers for family carers?			
