# Supplementary material for: Dementia care and the role of guideline adherence in primary care: cross-sectional findings from the DemTab study
Source: BMC Geriatr. 2021 Dec 18;21:717. doi: 10.1186/s12877-021-02650-8 (PMC8683809; doi:10.1186/s12877-021-02650-8)
Supplement: Supplementary file 2 — Additional file 2. Appendix 2. [file 12877_2021_2650_MOESM2_ESM.pdf]

Appendix 2

Table A.1. Comparison of Adherence to German Dementia Guideline (AGDG) scores across all scoring methods

AGDG score	*M*	*SD*	Median	Range
Scoring method 1	71.0	19.4	73.1	25 – 100
Scoring method 2	66.2	22.4	71.4	17 – 100
Scoring method 3	68.0	21.8	73.1	23 – 100
Scoring method 4	69.8	19.6	74.1	25 – 100

Note. *N* = 91, *M* = Mean, *SD* = Standard Deviation.

Table A.2. Correlations between AGDG scores across all scoring methods

AGDG score	1.	2.	3.
1. Scoring method 1	.		
2. Scoring method 2	.89**	.	
3. Scoring method 3	.92**	.99**	.
4. Scoring method 4	.97**	.96**	.98**

Note. ** $p < .01$.

Table A.3. Correlations between all scoring methods and other study variables

AGDG score	MMSE	FAST	ADL	IADL	QOL-AD	DIA-S	NPI
Scoring method 1	.24*	-0.09	.21*	0.10	0.07	-0.13	0.10
Scoring method 2	.28*	-0.10	.39**	0.19	0.13	-0.18	0.02
Scoring method 3	.27*	-0.10	.35**	0.19	0.12	-0.17	0.03
Scoring method 4	.26*	-0.09	.28**	0.15	0.11	-0.16	0.08

Note. MMSE = Mini Mental State Examination, FAST = Functional Assessment Staging, ADL = Barthel Index, IADL = Instrumental Activities of Daily Living, DIAS-S = Depression in Old Age Scale, NPI = Neuropsychiatric Inventory, EQ-5D-5L = Health Status, QOL- AD = Quality of Life Alzheimer's Disease. * $p < .05$. ** $p < .01$.
